# The Role of the Cytoskeletal Regulatory Protein, Mammalian Enabling Protein (Mena), in Invasion and Metastasis of HPV16-Related Oral Squamous Cell Carcinoma

**DOI:** 10.3390/cells13231972

**Published:** 2024-11-28

**Authors:** Zhichen Guo, Linyang Xie, Hao Cui, Xin Yang, Hong Qi, Ming Yu, Yuxin Gong, Junbo Tu, Sijia Na

**Affiliations:** 1 Key Laboratory of Shaanxi Province for Craniofacial Precision Medicine Research, College of Stomatology, Xi’an Jiaotong University, Xi’an 710004, China; guozc1988@xjtu.edu.cn (Z.G.); 15250032728@163.com (L.X.); ch021714@163.com (H.C.); 13380935031@163.com (X.Y.); 3122315512@stu.xjtu.edu.cn (M.Y.); charliegyx0223@163.com (Y.G.); 2 Department of Oral and Maxillofacial Surgery, College of Stomatology, Xi’an Jiaotong University, Xi’an 710004, China; 3 Department of Pathology, College of Stomatology, Xi’an Jiaotong University, Xi’an 710004, China; qihong@xjtu.edu.cn

**Keywords:** human papillomavirus 16, oral squamous cell carcinoma, Mena, epithelial–mesenchymal transition, metastasis

## Abstract

Background: The objective of this study was to investigate the effect of mammalian-enabled protein (Mena) on invasion and metastasis of HPV16-related oral squamous cell carcinoma (OSCC) and the underlying mechanism. Materials and methods: The Mena gene expression profile of HPV-related OSCC was analyzed from the TCGA, GEO and TIMER databases. Immunohistochemistry was performed to study Mena, and the expression of invasion and metastasis-related markers and their clinicopathological characteristics. The role of Mena in the biological behavior of OSCC cell lines was assessed through both non-transfected and stably transfected models, analyzing EMT-related markers in vitro. The effect of Mena on HPV16-related OSCC metastasis through immunodeficient mouse model in vivo. Results: Mena expression was significantly decreased in HPV16-positive OSCC, and Mena expression in HPV16-negative OSCC was related with lymphatic metastasis and TNM stages, and E-cadherin, vimentin and MMP-2, but it was not statistically significant in HPV16-positive OSCC. Increased Mena expression was significantly correlated with a poor overall survival and disease-free survival in an HPV16-negative OSCC patient. Mena plays a vital role in promoting OSCC cell migration, invasion and metastasis. Conclusions: Mena promotes OSCC invasion and metastasis in HPV-negative OSCC by activating the EMT process. However, Mena expression in OSCC infected with HPV16 is inhibited, thus suppressing its invasion and metastasis ability.

## 1. Introduction

Oral squamous cell carcinoma (OSCC) is a common type of head and neck cancer, and approximately 657,000 new cases of oral cancer and 330,000 deaths from oral cancer are reported globally each year [1]. The risk factors of OSCC include smoking, alcohol consumption and human papillomavirus (HPV) infection. Among this group of etiological factors, a HPV infection causes approximately 2–8% of OSCC cases. HPV16 is the most prevalent type of HPV involved in HPV-related cancers [2,3]. A HPV infection not only contributes to the occurrence of cancer, but also promotes cancer development. Furthermore, a HPV infection affects cancer proliferation, cancer stem cell development, cancer invasion and metastasis through oncoproteins expressed by HPV itself, such as E6/E7, and thus promotes the development of many types of cancers [4,5]. Notably, HPV-related head and neck squamous cell carcinoma (HNSCC) is a subgroup of head and neck cancers with a unique epidemiologic, clinical and molecular features. Previous studies have shown that HPV-positive HNSCC and oropharyngeal squamous carcinoma (OPSCC) have higher survival rates and a better prognosis than HPV-negative HNSCC and OPSCC [6,7]. Furthermore, in HNSCC, HPV can inhibit tumor invasion and metastasis by promoting FcGBP protein expression and modulating PRKCZ hypermethylation to inhibit epithelial–mesenchymal transition (EMT)-related pathways [8,9]. Additionally, HPV-positive HNSCC-derived exosomal miR-9-5p inhibits cancer-related fibroblast phenotypic transformation mediated by TGF-β signaling via NOX4 [10]. Although OSCC is a subtype of HNSCC, conclusions based on the research of HPV16-infected patients with HNSCC and OSCC are not entirely consistent, and many findings from prognostic studies of HPV-related OSCC are contradictory. Thus, the precise mechanism by which HPV16 infection regulates invasion and metastasis in OSCC remains largely unknown.

Mammalian-enabling protein (Mena) is a cytoskeletal regulatory protein from the ENA/VASP family encoded by the ENAH gene [11,12]. Mena plays a role in regulating the invasion and metastasis of multiple cancers. In particular, in breast cancer, Mena expression is significantly greater in cancer tissues than in normal breast tissues [13], and a decrease in Mena expression levels can reduce tumor metastasis [14]. In our previous studies, we reported that Mena can regulate the invasion and metastasis of OSCC by modulating the EMT process [15]. Moreover, previous research has suggested that Mena expression is regulated by HPV in OSCC [16]. On the basis of the results of our preliminary study, we performed bioinformatics analysis of data from The Cancer Genome Atlas (TCGA) and the Gene Expression Omnibus (GEO) and discovered that a HPV infection and Mena expression are negatively correlated in both HPV-related HNSCC and OSCC. However, the relationships among HPV16 infection, Mena protein levels, and OSCC invasion and metastasis remain unclear.

Therefore, the objectives of this study were to analyze Mena protein expression levels and the clinicopathological features of the Mena protein in patients with HPV16-related OSCC and to investigate the correlations among HPV16 infection, Mena protein levels, and OSCC invasion and metastasis via in vivo and in vitro experiments.

## 2. Materials and Methods

### 2.1. Bioinformatics Analysis

In the present study, Mena expression data from HPV-positive and HPV-negative OSCC samples were downloaded from TCGA (https://portal.gdc.cancer.gov/, accessed on 14 April 2022) and the GEO database (GSE40774, http://www.ncbi.nlm.nih.gov/geo/, accessed on 14 April 2022). The HPV Induced Cancer Resource (THInCR) (https://thincr.ca/, accessed on 14 April 2022) was used to differentiate patients with OSCC from which TCGA data were obtained by the HPV status. The TIMER (https://cistrome.shinyapps.io/timer/, accessed on 14 April 2022) online database was used to analyze the differential expression of ENAH (which encodes the Mena protein) between tumor tissues and adjacent normal tissues in all TCGA data with the Gene DE module.

### 2.2. Clinical and Pathological Assessments

Between 2013 and 2020, a total of 130 OSCC samples were obtained from the Stomatological Hospital affiliated with Xi’an Jiaotong University. Informed consent was obtained from all individuals who participated in this research. The inclusion criteria were as follows: (1) a diagnosis of OSCC, (2) a history of radical surgery, (3) the absence of other malignant tumors, and (4) available complete clinical records and follow-up information. Clinical evaluations and histopathological or cytological analyses were used to validate the HPV status and diagnose OSCC. The Medical Ethics Committee at Xi’an Jiaotong University’s Stomatological Hospital conducted a review and granted approval for the study (reference number: xjkqll [2022] no. 028). To evaluate the overall survival and disease-free survival rates, the Kaplan-Meier analysis of a cohort of 82 individuals, comprising 60 patients with HPV positivity and 22 without HPV, was conducted.

### 2.3. Immunohistochemical Staining

Immunohistochemical (IHC) staining was conducted with the Leica Bond MAX from Leica Microsystems (Weisar, Wetzlar, Germany), an automatic immunostaining device. After deparaffinization and rehydration, the sections were treated with Tris-EDTA buffer (pH 9.0) to facilitate antigen retrieval and subsequently subjected to a 20 min blocking process with 3% H_2_O_2_. Automated IHC staining was carried out with primary antibodies against Mena (ab244417, Abcam, Cambridge, UK, 1:100), P16INK4A (10883-1-AP, Sanying, Wuhan, China), E-cadherin (MAB-0738, Maixin, Fuzhou, CHN), vimentin (MAB-0735, Maixin, Fuzhou, China), and MMP-2 (ab37150, Abcam, Cambridge, UK, 1:500) and the Bond Polymer Refine-HRP Detection kit (DS9800, Leica, Newcastle, UK). This processed included incubation with a secondary antibody, development with DAB, and counterstaining with haematoxylin. The stained slides were assessed and evaluated via the IHC profiler technique with ImageJ software (latest v. 1.54v). The tissues were divided into four categories on the basis of the IHC staining results: high-positive (3+), positive (2+), low-positive (1+), and negative (0). High expression was indicated by an IHC score of 2 or more, whereas scores below this threshold indicated low expression. In each instance, three distinct areas were chosen for examination at a magnification of 200×, where IHC scores were produced using the IHC profiler, and the average score for each sample was computed.

### 2.4. Cell Culture and Cell Transfection

The human HPV16-negative OSCC cell line Cal27 was generously provided by the Department of Oral and Maxillofacial Surgery at Nanchang University (Nanchang, China) and was cultured in Dulbecco’s modified Eagle’s medium (Gibco, Thermo Fisher, Waltham, MA, USA). The human HPV16-positive OSCC cell line SCC090 was purchased from the Cell Bank of the Type Culture Collection of the Chinese Academy of Sciences (Shanghai, China). The cells were maintained in DMEM supplemented with 10% exosome-free FBS and maintained at 37 °C in a humidified environment containing 5% CO_2_. Genechem (Shanghai, China) produced lentiviruses containing a siRNA designed to target Mena (denoted as siMena) and lentiviruses that specifically express Mena (denoted as LV-Mena). Cal27 cells were cultured in 6-well plates at a density of 1 × 10^5^ cells per well and transfected with siMena following the guidelines provided by the manufacturer. Similarly, SCC090 cells were inoculated into 6-well plates at the same density and transduced with LV-Mena using an identical protocol. The cells were subjected to lentiviral infection for 12 to 16 h. Seventy-two hours after transfection, the viability of the cells was evaluated, and stable cell lines were established with puromycin. The transfection efficiency was evaluated by determining the percentage of cells displaying green fluorescence relative to the total number of adhered cells in 10 randomly chosen fields for each sample, and observations were conducted using fluorescence microscopy at a magnification of 100×. For subsequent analysis, Cal27 cells were categorized into two distinct groups: the control group (untransfected) and the siMena group (treated with siMena). Similarly, SCC090 cells were categorized into two experimental groups, the untransfected control group and the LV-Mena-transduced group, for further investigation.

### 2.5. Cell Invasion and Migration Assays

Invasion and migration assays were carried out with Cal27 cells sourced from both the control and siMena groups and SCC090 cells from the control and Mena-expressing groups. The invasion assay was carried out in a Transwell system, which consists of a polycarbonate membrane featuring 8-micrometre pores (Corning, Shanghai, China) situated in a 6-well plate. A coating of 50 µL of Matrigel Basement Membrane Matrix (BD Biosciences, Bedford, UK) was applied to the Transwell insert. The cells were resuspended in 500 μL of DMEM lacking FBS and introduced into the Transwell insert. In the lower chamber, 1.5 mL of DMEM supplemented with 10% FBS was added, and the cells were incubated at 37 °C as they invaded through the Matrigel. After 48 h, the cells located on the lower side of the Transwell insert were preserved in 75% methanol and subsequently stained with a 0.5% (*m*/*v*) crystal violet solution. Observations were made using an inverted microscope (Olympus FSX100, Olympus, Tokyo, Japan), and the ability of the cells to invade was assessed via ImageJ software. A migration assay was conducted in a manner similar to the invasion assay, with the only difference being that the Transwell insert lacked a coating. Each trial was conducted with three repetitions.

### 2.6. Assessment of Wound Healing Capacity

Cal27 cells from both the control and siRNA-treated groups and SCC090 cells from both the control and the Mena-expressing groups were seeded in 6-well plates at a density of 5 × 10^5^ cells per well and incubated at 37 °C with 5% CO_2_ for one night. When the cell coverage reached approximately 90%, a sterile pipette tip was used to create a scratch. Following the formation of the wound, the cells were incubated for an additional 24 h. Photographs were taken with an Olympus FSX100 inverted microscope, and cellular motility was analyzed via Image-Pro Plus software (latest v. 7.0).

### 2.7. Western Blotting

Proteins were extracted from cells using a RIPA Buffer Kit (ZHHC, Xi’an, China). The protein concentration was assessed with a BCA Protein Assay Kit (BOSTER, Wuhan, China). PAGE gels were subsequently prepared with a Colour Gel Rapid Preparation Kit (ZHHC, Xi’an, China). A total of 20 μg of protein was denatured in a water bath, separated by SDS-PAGE, and transferred to a PVDF membrane, which was then blocked with TBST containing 5% skim milk for 2 h. Primary antibodies against the following were used: P16INK4A (10883-1-AP, Sanying, Wuhan, China), Mena (ab244417, Abcam, Cambridge, UK, 1:100), MMP-2 (ab37150, Abcam, Cambridge, UK, 1:500), Snail + Slug (bs-11961R, BIOSS, Beijing, China, 1:500), E-cadherin (bs-1519R, BIOSS, Beijing, China, 1:500), N-cadherin (ab37150, Abcam, Cambridge, UK, 1:500) and GAPDH (BM3874, BOSTER, Wuhan, China, 1:500). After incubation overnight at 4 °C, secondary antibodies (bs-11961R; BIOSS, Beijing, China; 1:500) were added to the membrane. Finally, 200 μL of a luminescent reagent (AR1197, BOSTER, Wuhan, China) was added, the mixture was loaded into a Bio-Rad ChemiDoc XRS system, and the protein expression levels were assessed via Image Lab software (latest v. 4.1).

### 2.8. In Vivo Experiments to Evaluate Tumour Metastasis

The animal study received approval from the Institutional Animal Care and Use Committee at the Health Sciences Center of Xi’an Jiaotong University (ethics code: XJTUAE-2014-1536). Four-week-old immunocompromised mice were obtained from the Laboratory Animal Center within the Xi’an Jiaotong University Health Sciences Center. To establish a model of tumor metastasis, Cal27 cells were classified into two groups: the control group and the siMena group. Moreover, SCC090 cells were classified into the control and Mena-expressing groups. These cells were then injected into the lateral tail vein of random anaesthetized immunodeficient mice at a concentration of 2 × 10^5^ cells in 100 μL of PBS. The mice were marked, and each cohort included four subjects. Four weeks later, all the mice were euthanized via carbon dioxide asphyxiation. The livers were collected for staining with hematoxylin and eosin and IHC evaluation. Liver metastases were assessed by counting the tumor foci in five randomly chosen fields of view.

### 2.9. Statistical Analysis

All the statistical analyses were conducted using SPSS Statistics 24.0 (IBM, Armonk, NY, USA). The χ^2^ test was used to assess the correlation between Mena expression levels and clinicopathological characteristics or tumor-related markers. Student’s *t*-test was used for further statistical comparisons. The data are presented as the means ± SDs from at least three independent experiments. Statistical significance is indicated as follows: * *p* < 0.05, ** *p* < 0.01, and *** *p* < 0.001.

## 3. Results

### 3.1. Mena Expression Is Higher in HPV16-Negative OSCC than in HPV16-Positive OSCC and Is Correlated with Patient Survival

**Figure 1:**
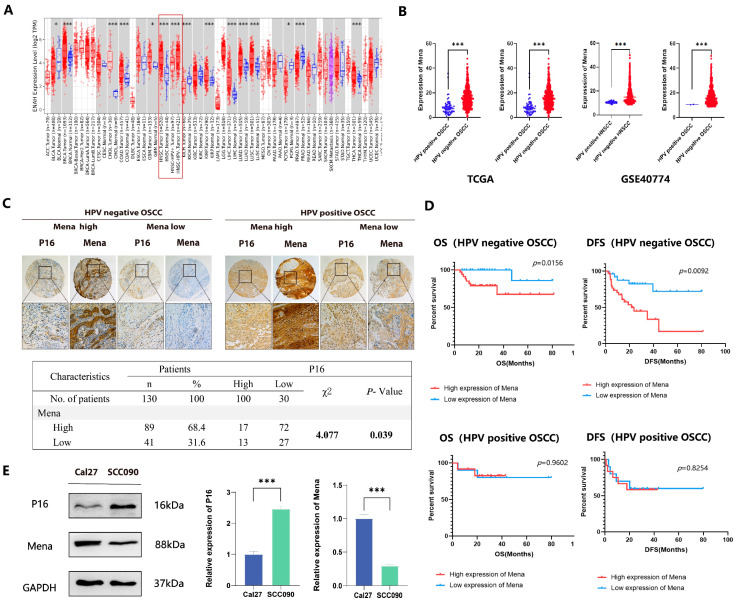
The abnormal expression of Mena mRNA between HPV16-positive and -negative OSCC. (**A**) The TIMER platform represents the expression of Mena mRNA in malignancies and normal tissues based on the TCGA database. (**B**) Mena expression in HPV-positive and -negative HNSCC and OSCC in TCGA and GEO (GSE40774) databases. (**C**) Immunohistochemistry of Mena protein and tumor-related marker expression in HPV16-negative and -positive OSCC. (**D**) Kaplan–Meier survival curves for Mena expression in HPV-positive and -negative OSCC patients. (**E**) Differential Mena expression in a HPV16-negative sexual cancer cell line Cal27 and HPV-positive cancer cell line SCC090 (* *p* < 0.05, *** *p* < 0.001).

After RNA sequencing, data reflecting Mena expression in different malignant tumors from the TCGA database were compared with the TIMER platform, our analysis revealed that Mena expression was greater in most tumor tissues (including HNSCC tissues) than in normal tissues. Furthermore, we observed significantly higher Mena expression levels in HPV-negative HNSCC than in HPV-positive HNSCC (Figure 1A). We then obtained data from patients with HNSCC and OSCC from the TCGA database and GSE40774, categorized the patients on the basis of HPV status, and compared Mena expression levels in the patients, which indicated that Mena expression levels were significantly greater in HPV-negative cancers than in HPV-positive cancers (both HNSCC and OSCC, *p* < 0.001), which is consistent with the results above (Figure 1B). To further assess the expression of Mena in HPV16-related OSCC, IHC staining of 130 OSCC tissues was performed after categorizing the OSCC patients into 30 HPV16-positive and 100 HPV16-negative patients on the basis of P16INK4A (P16) staining and classifying the patients on the basis of Mena expression determined by IHC staining into high and low Mena expression groups. The results revealed that Mena expression levels were greater in HPV16-negative cancers than in HPV16-positive cancers (*p* = 0.039) (Figure 1C). Moreover, Kaplan-Meier survival analysis revealed that Mena expression levels were related to patient survival only in patients with HPV16-negative cancers and that patients with high Mena expression had lower overall survival (*p* = 0.016) and disease-free survival (*p* = 0.009) rates, whereas there was no significant correlation between Mena expression levels and patient survival in patients with HPV16-positive cancers (Figure 1D). These results were further validated in HPV16-related OSCC cell lines via Western blot experiments, which revealed higher Mena protein expression levels in the HPV16-negative OSCC cell line Cal27 than in the HPV16-positive OSCC cell line SCC090 (*p* < 0.001) (Figure 1E).

### 3.2. HPV16 Inhibits OSCC Invasion and Metastasis by Suppressing Control of the EMT Process by Mena

**Figure 2:**
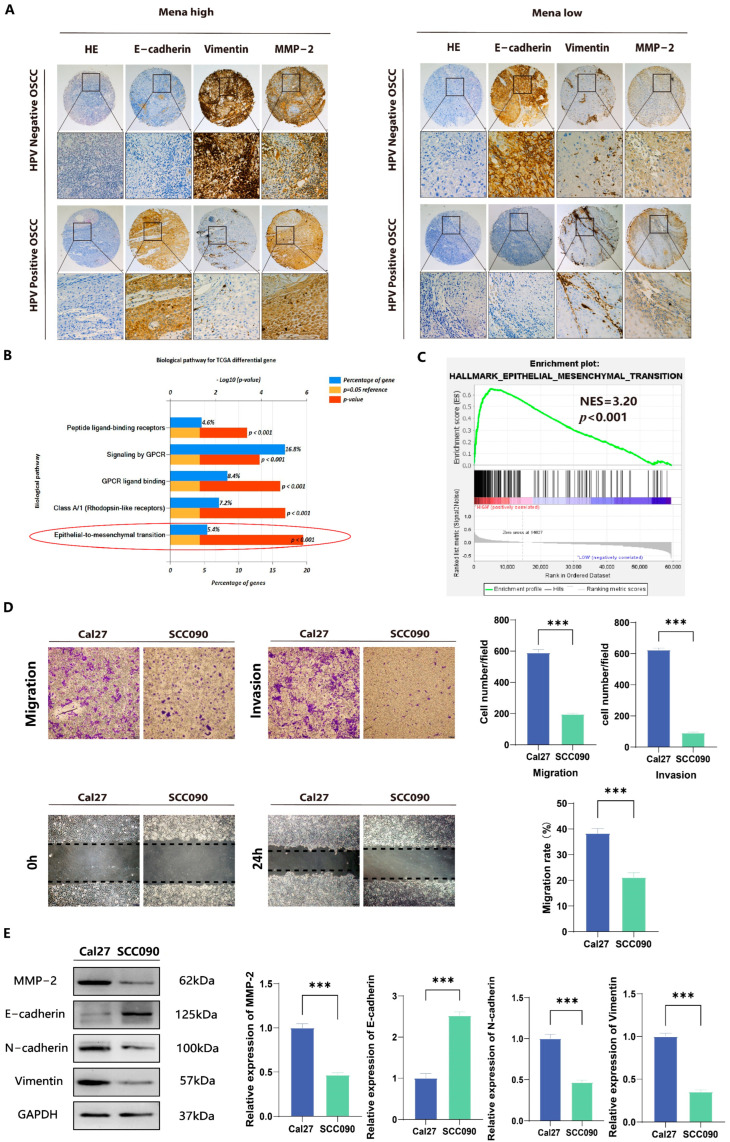
Association of Mena and EMT in HPV16-positive and -negative OSCC. (**A**) Immunohistochemical analysis of Mena protein and tumor-related marker expression in HPV16-positive and -negative OSCC. (**B**) Biopathology enrichment of differential genes for HPV-positive and -negative cancers in the TCGA database by Funrich. (**C**) GSEA analysis to investigate the potential regulatory mechanisms with tumor hallmarks. (**D**) Representative images show that Mena’s cells in the HPV16-negative cancer cell line Cal27 have a significantly higher migration and invasion capacity than the HPV16-positive cancer cell line SCC090. (**E**) Western blotting was performed to measure the protein expression levels of Mena, MMP2, E-cadherin and vimentin of Cal27 and SCC090 cells (*** *p* < 0.001).

Previously collected data suggest that Mena overexpression plays a role in the poor prognosis of OSCC and OSCC progression by facilitating the EMT process. To explore the role of Mena in HPV16-related OSCC, the correlation of Mena expression levels with clinicopathological factors was first assessed. In HPV16-negative OSCC, Mena expression was significantly correlated with lymph node metastasis (χ^2^ = 5.448, *p* = 0.020) and TNM stage (χ^2^ = 4.702, *p* = 0.030) (Table 1). However, in HPV16-positive OSCC, Mena expression did not correlate with any clinicopathological factors (Table 2). Moreover, in HPV16-negative OSCC, high Mena expression levels were related to low expression levels of the EMT-related protein E-cadherin (χ^2^ = 4.128, *p* = 0.042), high expression levels of the EMT-related protein vimentin (χ^2^ = 4.834, *p* = 0.028), and high expression levels of the invasion-related protein MMP-2 (χ^2^ = 8.130, *p* = 0.004) (Figure 2A, Table 1), whereas there were no significant correlations in HPV16-positive OSCC (Figure 2A, Table 2). Moreover, we found by pathway analysis of differentially expressed genes obtained from the TCGA database and GSEA pathway enrichment analysis of Mena expression data (Figure 2B,C) that both HPV status and Mena expression levels were related to the EMT process, which is consistent with our clinicopathological results. The same results were obtained in HPV16-related OSCC cell lines; the invasive and migratory capacity of Cal27 cells was significantly greater than that of SCC090 cells (*p* < 0.001) (Figure 2D), whereas the protein expression levels of E-cadherin, vimentin and MMP-2 in Cal27 cells were significantly greater than those in the latter cell line (*p* < 0.001) (Figure 2E). In conclusion, HPV16 infection inhibited the expression of Mena, and in HPV16-negative cancers, high Mena expression levels activated the EMT process, which promoted tumor invasion and metastasis.

### 3.3. HPV16 Infection Suppresses OSCC Invasion and Metastasis by Inhibiting the Ability of Mena to Mediate Snail- and Slug-Regulated EMT

**Figure 3:**
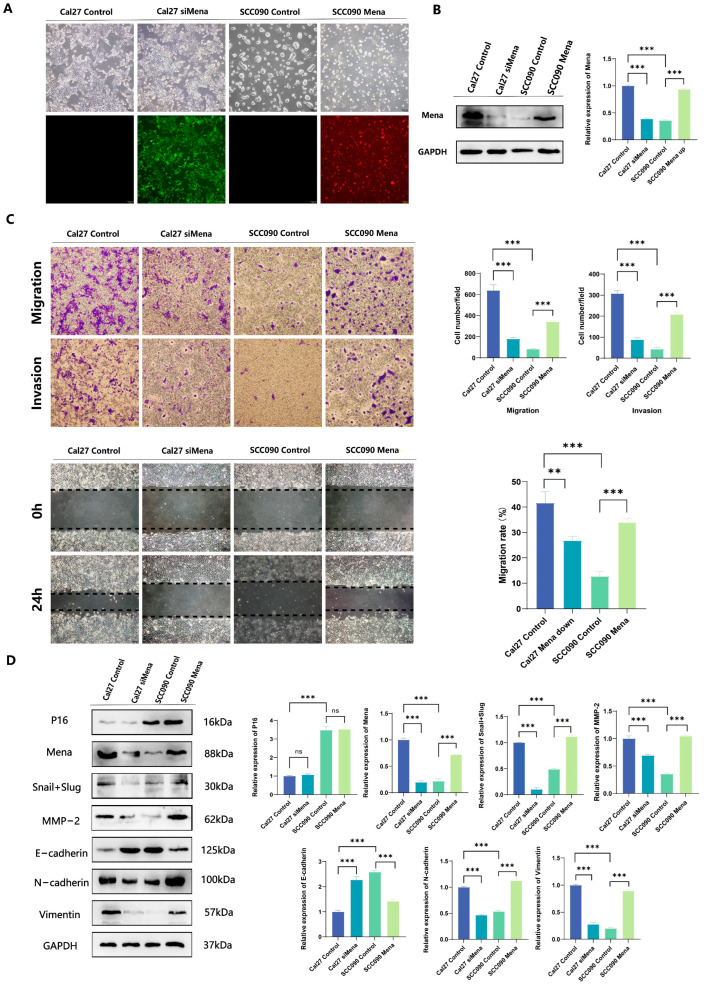
The impact of Mena on cell migration and invasion in Cal27 and SCC090 cells. (**A**,**B**) Cal27 cells were transfected with siMena, while SCC090 cells were transfected with Mena; successful transfection was confirmed by fluorescence microscopy and Western blotting. (**C**) Representative images demonstrate that Mena enhances cell migration and invasion. (**D**) Western blotting was conducted to evaluate the protein expression levels of P16, Mena, MMP2, Snail, Slug, E-cadherin, N-cadherin and vimentin in Cal27 cells from the control and siMena groups, as well as in SCC090 cells from the control and Mena groups (** *p* < 0.01, *** *p* < 0.001).

To further validate the relationship between HPV16 infection and the ability of Mena to mediate the EMT process, we knocked down the Mena protein in Cal27 cells, overexpressed the Mena protein in SCC090 cells, and verified the changes in Mena protein expression via fluorescence microscopy and Western blotting (*p* < 0.001) (Figure 3A,B). The results of invasion and metastasis assays in the four groups suggested that the knockdown of Mena in Cal27 cells significantly decreased invasion and metastasis capacity (*p* < 0.001) (Figure 3C). Moreover, we re-examined the expression of EMT-related proteins and found that when Mena was knocked down, the expression levels of the EMT-switching proteins Snail and Slug decreased, the expression levels of the epithelial marker E-cadherin increased, the expression levels of the mesenchymal markers N-cadherin and vimentin decreased, and the expression levels of the invasion-related protein MMP-2 decreased (*p* < 0.001) (Figure 3D). In contrast, when Mena was overexpressed in SCC090 cells, invasion and metastatic ability was significantly elevated (*p* < 0.001) (Figure 3C), whereas the expression levels of the EMT switching proteins Snail and Slug were increased. The expression level of E-cadherin decreased, whereas the expression levels of N-cadherin, vimentin and MMP-2 increased (*p* < 0.001) (Figure 3D). These findings are consistent with our previous conclusion that HPV16 infection inhibits the EMT process by suppressing the expression of Mena and thus the expression of the EMT switching proteins Snail and Slug, resulting in decreased invasion and metastatic ability in OSCC.

### 3.4. The Roles of Mena in the Progression and Metastasis of OSCC in Live Models

**Figure 4:**
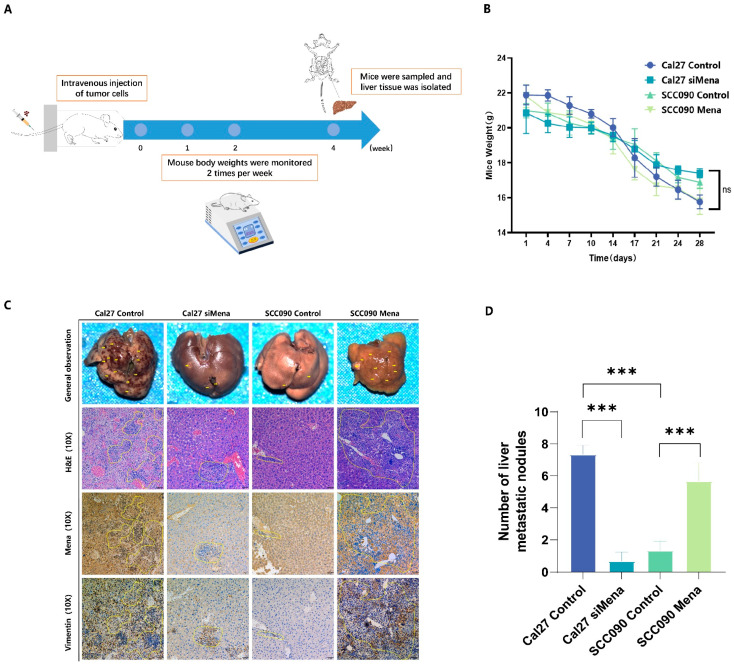
Effect of Mena on tumor metastasis in Cal27 and SCC090 cells in immunodeficient mice. (**A**) Overview of the mouse tail vein injection procedure. (**B**) Changes in body weight of mice 4 weeks postinjection. (**C**,**D**) Representative images and analysis of liver metastasis lesions from OSCC in the Cal27 control and siMena groups, as well as in the SCC090 control and Mena groups (*** *p* < 0.001; Magnification: ×100).

To investigate the impact of Mena on HPV16 infection-related metastasis in OSCC, immunodeficient mice were injected with control Cal27 cells, Cal27-siMena cells, control SCC090 cells, or SCC090-Mena cells. Changes in the weight of the mice were monitored for four weeks postinjection, and metastatic HPV16-related OSCC nodules on the liver surface of the mice were excised, stained with HE for immunohistochemistry, and analyzed after four weeks (Figure 4A,B). The HPV16-negative OSCC nodules demonstrated a significant decrease in tumor metastasis upon Mena knockdown (*p* < 0.001), while the HPV16-positive OSCC nodules showed a substantial increase in tumor metastasis with greater Mena levels (*p* < 0.001) (Figure 4C,D). Moreover, the expression levels of EMT-related protein vimentin underwent similar changes when Mena was knocked down or overexpressed (Figure 4C), indicating that a HPV16 infection alters Mena expression to control the EMT process in vivo.

## 4. Discussion

A substantial amount of epidemiological evidence indicates that a HPV infection may contribute to various types of human cancer. Malignant tumors are highly linked to 12 types of HPVs, with HPV16 being the most prevalent type [6]; infection with these types of HPVs causes a range of cancers, such as cervical and breast cancer [17,18]. HPV-related cancers are characteristically stimulated by the oncoproteins of HPV16, which promote cancer growth, invasion and metastasis through multiple mechanisms. In contrast, HPV16-related HNSCC has epidemiologic, clinical and molecular profiles that are distinct from those of other cancers [19]. Compared with patients with HPV16-negative cancers, patients with HPV16-positive cancers react better to cancer treatments, such as surgery, radiotherapy, and chemotherapy, resulting in lower invasion and metastasis rates and a better prognosis [20]. This outcome has been verified in OPSCC [21]. However, whether HPV16 can also regulate the invasion and metastasis of OSCC has remained inconclusive, and more in-depth studies are still needed.

A HPV infection and Mena protein levels are closely associated in OSCC [16]. Mena, a regulatory cytoskeletal protein, plays critical roles in actin-dependent cell motility, cell adhesion, actin network assembly, and regulation [11,22]. Mena expression is upregulated in various malignant tumor tissues relative to normal tissues, which promotes invasion and metastasis [23]. In this study, we examined the connection between HPV16 infection and Mena protein expression, and the results revealed that Mena expression levels are elevated in HPV-negative patients with either HNSCC or OSCC. Then, after categorizing 130 OSCC patients into HPV16-positive and HPV16-negative cancer patients and evaluating differences in Mena expression, we found that Mena expression levels were greater in most HPV16-negative OSCC patients. Our previous research indicated that OSCC patients with high Mena expression exhibit a more advanced TNM stage and have more lymph nodes, ultimately resulting in poorer disease-free survival and overall survival [24]. An investigation of the relationships between clinicopathologic features and Mena expression in HPV16-related OSCC revealed that Mena expression was related to lymph node metastasis and TNM stage only in patients with HPV16-negative OSCC and that patients with HPV16-negative OSCC with high Mena expression levels had increased lymph node metastasis and exhibited a more advanced TNM stage with a poorer prognosis, which is consistent with previous research. Interestingly, in patients with HPV-positive OSCC, the expression of Mena did not impact lymph node metastasis, TNM stage, or OSCC prognosis. This may be because a HPV16 infection simultaneously inhibits Mena expression and inhibits the effects of Mena on downstream invasion- and metastasis-related pathways.

In recent years, numerous studies have shown that EMT is the primary mechanism through which epithelial cancer cells acquire a malignant phenotype, causing invasion and metastasis [25]. Mena has been shown to promote OSCC invasion and metastasis by activating the EMT process [24]. Further research revealed that Mena is highly expressed in HPV16-negative OSCC, that high Mena expression is related to the EMT process, and that high Mena expression inhibits E-cadherin expression and promotes N-cadherin, vimentin, and MMP-2 expression. In contrast, in HPV16-positive OSCC, Mena expression levels are reduced, and the EMT process is inhibited. E-cadherin, a crucial apical junction complex, is a transmembrane protein that anchors neighboring cells together. A reduction in E-cadherin levels is necessary for the EMT process [26]. Vimentin, a vital EMT marker, is widely expressed in mesenchymal cells and mesenchymal-derived cells, and participates in maintaining cellular integrity and promoting cell migration and invasion during EMT [27]. Furthermore, MMP-2 plays a pivotal role in the degradation of the extracellular matrix (ECM) during cancer progression, inducing cancer cells to migrate beyond the primary tumor to create metastatic foci. Additionally, MMP-2 triggers EMT by activating the TGF-β pathway [28]. In summary, HPV16 inhibits the EMT process by suppressing Mena expression, which inhibits the invasion and metastasis of OSCC. Previous research revealed that Snail and Slug initiate EMT and that Smad pathway-mediated Snail and Slug transcription stimulates EMT by downregulating E-cadherin expression and upregulating N-cadherin and vimentin expression [29]. The results of in vitro experiments showed that the transfection of Cal27 cells with siMena inhibited the EMT process by suppressing Snail and Slug transcription, thereby decreasing the expression of MMP-2, resulting in significant decreases in invasion and metastasis. Similarly, the results of in vivo experiments revealed fewer hepatic metastases and lower expression levels of Mena and vimentin in the group injected with siMena-transfected Cal27 cells. The results of related studies demonstrated that Mena is involved in the EMT process in a variety of cancers, including liver, breast and gastric cancers. Moreover, Mena is involved in the regulation of cytoskeletal dynamics during EMT, which enhances the metastasis and invasiveness of cancer cells, consistent with the results of the present study [30]. Moreover, the results of in vitro experiments revealed that the EMT process was enhanced in the control SCC090 cells compared with the SCC090 cells overexpressing Mena due to the increased initiation of Snail and Slug transcription and upregulation of the expression of MMP-2, which resulted in significantly increased invasion and metastasis. Furthermore, the results of in vivo experiments revealed that the group injected with SCC090 cells overexpressing Mena had more liver metastases and higher expression levels of Mena and vimentin. Taken together, these findings indicate that Mena enables OSCC invasion and metastasis by activating Snail and Slug transcription, thereby promoting the EMT process and increasing the expression of MMP-2. However, the expression of Mena is suppressed in HPV16-positive OSCC, which inhibits this mechanism.

To our knowledge, this is the first study to find that Mena may serve as a mediator of HPV16 infection that inhibits OSCC invasion and metastasis. In HPV16-positive OSCC, Mena protein expression is inhibited; thus, Mena cannot control the EMT switching proteins Snail and Slug, and eventually, E-cadherin cannot be converted to N-cadherin. This effect suppresses the expression of vimentin, which inhibits tumor invasion and migration. However, due to the limitations of the present study, the pathway through which HPV regulates Mena expression remains unknown. A previous study suggested that the TGF-β pathway may play a role in this regulatory mechanism, as HPV can inhibit the TGF-β pathway in HNSCC patients and increases production of the FCGBP protein, which is correlated with increased survival time [8]. Furthermore, in breast cancer, hypoxia-induced activation of the TGF-β-RBFOX2-ESRP1 axis can regulate the alternative splicing of human Mena and promote the EMT process [30]. On the basis of these findings, we conducted a preliminary exploration via the bioinformatics analysis of OSCC patient data from the TCGA database and found that TGF-β and Mena expression levels were significantly and positively correlated in both HPV-positive and HPV-negative OSCC patients. However, whether TGF-β regulates the expression of Mena in HPV16-related OSCC is not clear, so further studies are needed to verify the underlying molecular mechanism involved. Furthermore, the immune microenvironment plays a crucial role in regulating tumor development and has become a significant focus in cancer research. The Mena protein may be pivotal in this context, however, the relationship between Mena and immune response, as well as its potential as a target for tumor immunotherapy, remains unclear. This will also be the primary focus of our forthcoming research.

## 5. Conclusions

In summary, this investigation has revealed an increase in Mena expression in OSCC patients that are negative for HPV. Increased Mena expression is correlated with both lymphatic spread and the TNM stage of the tumor, but these correlations were not observed in patients that are positive for HPV. Mena expression significantly increases the invasion and metastatic capacity of OSCC, which facilitates the transition of an epithelial cell phenotype to a more mesenchymal phenotype. These results suggest that the cytoskeletal protein Mena may be a potential prognostic biomarker for HPV16-related OSCC, providing a theoretical basis for the development of molecular targeted therapies and immunotherapies for patients with HPV16-related OSCC.

## Figures and Tables

**Table 1:** The relationship between the Mena expression and clinicopathological characteristics of 100 cases of HPV16-negative OSCC patients.

Characteristic	Patients		Mena			
	n	%	High	Low	χ^2^	*p*-Value
No. of patients	100	100	72	28		
gender						
Male	58	58	45	13	1.606	0.205
Female	42	42	27	15		
Age						
>60 years	61	61	45	16	1.057	0.304
≤60 years	39	39	27	12		
Tumor recurrence						
Yes	36	36	29	7	0.031	0.861
No	64	64	43	21		
Lymphatic metastasis						
Yes	65	65	54	11	5.448	0.02
No	35	35	18	17		
Tumor grade						
high	76	76	55	21	0.292	0.589
low	24	24	17	7		
TNM stage						
I/II	27	27	13	14	4.702	0.03
III/IV	73	73	59	14		
E-cadherin						
High	33	33	19	14	4.128	0.042
Low	67	67	53	14		
Vimentin						
High	41	41	36	5	4.834	0.028
Low	59	59	36	23		
MMP-2						
High	78	78	63	15	8.13	0.004
Low	22	22	9	13		

**Table 2:** The relationship between the Mena expression and clinicopathological characteristics of 30 cases of HPV16-positive OSCC patients.

Characteristic	Patients		Mena			
	n	%	High	Low	χ2	*p*-Value
No. of Patients	30	100	17	13		
gender						
Male	22	73	11	11	1.493	0.212
Female	8	27	6	2		
Age						
>60 years	14	47	7	7	0.475	0.374
≤60 years	16	53	10	6		
Tumor recurrence						
Yes	9	30	6	3	0.524	0.377
No	21	70	11	10		
Lymphatic metastasis						
Yes	20	67	13	7	1.697	0.181
No	10	33	4	6		
Tumor grade						
High	14	47	9	5	0.621	0.339
Low	16	53	8	8		
TNM stage						
I/II	7	23	2	5	2.935	0.101
III/IV	23	77	15	8		
E-cadherin						
High	12	40	5	7	1.833	0.164
Low	18	60	12	6		
Vimentin						
High	13	43	9	4	1.475	0.200
Low	17	57	8	9		
MMP-2						
High	24	80	15	9	1.663	0.204
Low	6	20	2	4		

## Data Availability

The raw data supporting the conclusions of this article will be made available by the authors on request.
